# Continuous Glucose Monitoring Enabled by Fluorescent Nanodiamond Boronic Hydrogel

**DOI:** 10.1002/advs.202203943

**Published:** 2023-01-16

**Authors:** Jian Zhang, Yongjun Zheng, Jimmy Lee, Alex Hoover, Sarah Ann King, Lifeng Chen, Jing Zhao, Qiuning Lin, Cunjiang Yu, Linyong Zhu, Xiaoyang Wu

**Affiliations:** ^1^ Ben May Department for Cancer Research University of Chicago Chicago IL USA; ^2^ Key laboratory for Advanced Materials and Joint International Research Laboratory of Precision Chemistry and Molecular Engineering Feringa Nobel Prize Scientist Joint Research Center School of Chemistry and Molecular Engineering East China University of Science and Technology Shanghai 200237 China; ^3^ Burns Center of Changhai Hospital Shanghai China; ^4^ Pritzker School of Molecular Engineering University of Chicago IL USA; ^5^ School of Biomedical Engineering Shanghai Jiao Tong University800 Dong Chuan Road Shanghai 200240 China; ^6^ Departments of Engineering Science and Mechanics, Biomedical Engineering, Materials Science and Engineering Materials Research Institute Pennsylvania State University University Park PA 16802 USA

**Keywords:** boronic hydrogel, diabetes, fluorescent nanodiamond, glucose, microneedle

## Abstract

Continuous monitoring of glucose allows diabetic patients to better maintain blood glucose level by altering insulin dosage or diet according to prevailing glucose values and thus to prevent potential hyperglycemia and hypoglycemia. However, current continuous glucose monitoring (CGM) relies mostly on enzyme electrodes or micro‐dialysis probes, which suffer from insufficient stability, susceptibility to corrosion of electrodes, weak or inconsistent correlation, and inevitable interference. A fluorescence‐based glucose sensor in the skin will likely be more stable, have improved sensitivity, and can resolve the issues of electrochemical interference from the tissue. This study develops a fluorescent nanodiamond boronic hydrogel system in porous microneedles for CGM. Fluorescent nanodiamond is one of the most photostable fluorophores with superior biocompatibility. When surface functionalized, the fluorescent nanodiamond can integrate with boronic polymer and form a hydrogel, which can produce fluorescent signals in response to environmental glucose concentration. In this proof‐of‐concept study, the strategy for building a miniatured device with fluorescent nanodiamond hydrogel is developed. The device demonstrates remarkable long‐term photo and signal stability in vivo with both small and large animal models. This study presents a new strategy of fluorescence based CGM toward treatment and control of diabetes.

## Graphical abstract

Continuous glucose monitoring (CGM) allows diabetic patients to better maintain blood glucose level. In this proof‐of‐concept study, a skin mountable device is developed with fluorescent nanodiamond hydrogel, presenting a new strategy of fluorescence based CGM toward treatment and control of diabetes.

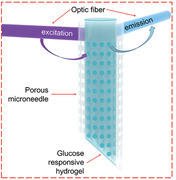

## 1 Introduction

Obesity and diabetes are an acute and growing public health problem around the world.^[^ ^1^ ^]^ The most common approach to detect blood glucose concentration for diabetic patients still relies on finger pricking for sample collection. However, recent study indicates that the compliance of this practice can be as low as 17.6% in diabetic patients, due to the invasiveness and pain associated with finger pricking. Noninvasive monitoring of blood glucose level in a continuous manner can provide significant clinical benefits to diabetic patients.^[^ ^2^, ^3^, ^4^, ^5^ ^]^ The current methodologies of continuous glucose detection mostly depend on electrochemical approaches (enzymatic or nonenzymatic approaches) and optical approach (fluorescence, fluorescence resonance energy transfer (FRET), ocular spectroscopy, infrared spectroscopy, photoacoustic spectroscopy, etc.).^[^ ^2^, ^5^, ^6^, ^7^, ^8^ ^]^ In order to sense the glucose level in vivo, most current technologies have to use invasive sensors that are implanted subcutaneously or intravenously. However, implantation of the devices can lead to significant tissue inflammation and biofouling‐induced sensor degradation, causing loss of sensitivity. Although less invasive approaches have been developed, such as iontophoresis, sonophoresis, smart tattoo, and microneedles, these technologies suffer from various drawbacks, including calibration inaccuracy, biological incompatibility, current‐induced skin erythema or irritation, significant subject‐to‐subject variability, and long warm‐up or duration time required to collect sufficient amount of sample.^[^ ^2^, ^4^, ^5^, ^6^, ^7^, ^8^, ^9^ ^]^

The current CGM systems approved by the Food and Drug Administration (FDA) include Dexcom G6, FreeStyle Libre 3 by Abbott, Medtronic Guardian 3, and Eversense CGM by Senseonics. The first three products are all based on electrochemical signal transduction with enzymatic amperometric electrode sensors, which are usually needle‐like filaments with a length of 5 to 10 mm. However, enzymes, such as glucose oxidase, used for the glucose sensing are not stable in vivo, due to protein degradation and aggregation. The enzymatic reaction produces hydrogen peroxide and other reactive free radicals, which can trigger foreign body reaction^[^ ^10^, ^11^ ^]^ and compromise the performance of biosensors.^[^ ^12^, ^13^, ^14^ ^]^ Glucose consumption by enzymatic reaction can also influence the glucose concentration in the surrounding tissue and thus affect the glucose diffusion gradient, lowering the reliability of the sensors. In addition, sensors with metal‐based electrodes heavily rely on the available surface area for direct oxidation, but the metal surface of the electrodes can be damaged by corrosion in vivo, and the electronic device is also vulnerable to the water permeation. Eversense E3 has a long duration (180 days) owing to its unique nonenzymatic fluorescent responsibility for blood glucose. However, the cylindrical sensor of Eversense E3 is bulky with a length of 18.3 mm and a diameter of 3.5 mm. Because of the size limitation, a surgical operation is required for implantation or displacement of the sensor. In addition, the formation of avascular fibrous scar around the implanted sensor and the subcutaneous tissue impedes the feasibility of long‐term implantation.^[^ ^15^, ^16^, ^17^ ^]^ The acute inflammatory response to tissue damage and presence of a large foreign body compromise the accuracy of the glucose sensors after implantation for multiple days.^[^ ^18^ ^]^ The Eversense E3 requires a long stabilization time with frequent calibrations throughout its lifetime. A more stable and user friendly CGM device has been long thought‐after and is extremely critical for diabetic patients to better monitor and manage their glucose level daily.

**Figure 1:**
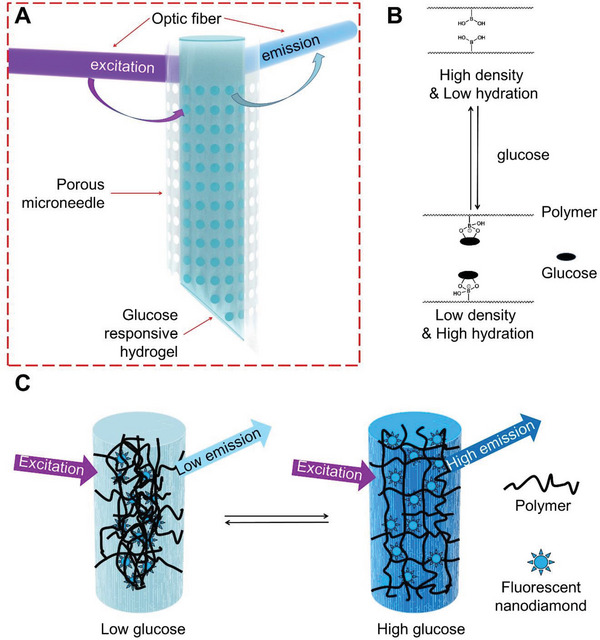
Schematics of the microneedle with fluorescent nanodiamond based hydrogel. A) Schematic illustration of the microneedle device comprising transparent porous wall and covalently bound fluorescent nanodiamond based boronic hydrogel for glucose sensing. B) The reversible complexation of boronic acid group and cis diols of glucose molecules enables the reversible changes of the polymer network at different glucose concentrations. C) Schematic illustration of the glucose‐responsive hydrogel that can change fluorescence output according to glucose concentration.

**Figure 2:**
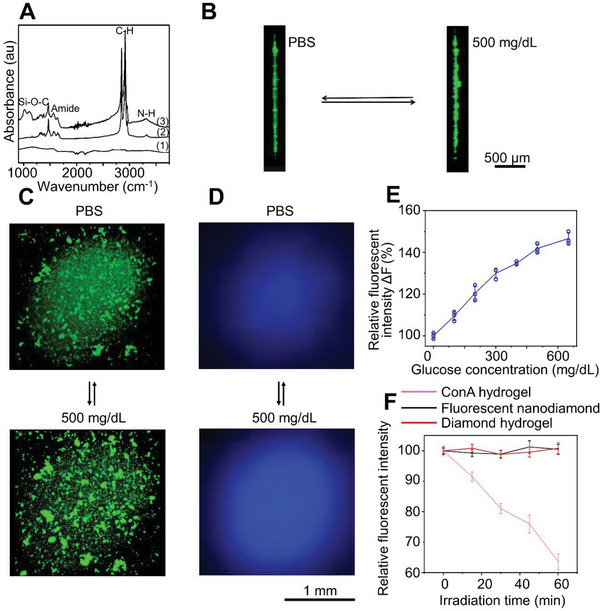
Fabrication of fluorescent nanodiamond hydrogel for glucose sensing. A) FTIR spectra of unmodified nanodiamond (1), octadecylamine modified nanodiamond (2), and octadecylamine modified nanodiamond with alkene surface group (3). The cross‐section (B) and top view (C) of the confocal images show that the glucose molecules can reversibly trigger the swelling of the hydrogel in vitro. D) In vitro fluorescence imaging shows that the nanodiamond hydrogel has higher fluorescent emission in the presence of glucose (500 mg dL^−1^) compared with phosphate‐buffered saline (PBS) solution. E) Fluorescent emission of the nanodiamond based hydrogel shows strong correlation with glucose concentration in vitro, *n* = 3. Data are presented as mean ± standard deviation (SD). All error bars represent SD. F) Compared with the classical Con A glucose‐responsive hydrogels, the nanodiamond based hydrogels exhibited excellent photostability in vitro, *n* = 4. Data are presented as mean ± SD. All error bars represent SD.

In this study, we report a fluorescent nanodiamond based device for CGM, comprising the following: (1) a microneedle with a transparent porous barrel and a fluorescent nanodiamond based boronic hydrogel covalently bound to internal barrel (**Figure** **1**); (2) a design of the device configured to transmit excitation and emission signals through the microneedle via optical fibers; (3) a data processor for retrieving and analysis of data from the optical sensor. Photobleaching is a significant issue for fluorescence imaging, particularly important for long‐term CGM based on fluorescent signals. Compared with other fluorophores, such as quantum dots and organic dyes, fluorescent nanodiamond has lower toxicity, exceptional chemical stability, biocompatibility, outstanding photostability, and facile surface for modification. When functionalized by organotrialkoxysilane with alkene terminal groups, we can covalently integrate fluorescent nanodiamond into the phenylboronic acid functionalized hydrogel for glucose sensing. To circumvent the issue associated with full device implantation, we designed and successfully loaded and integrated the fluorescent nanodiamond based boronic hydrogel to porous microneedles, which have randomly distributed but interconnected pores. The porous structure can effectively enhance extraction of interstitial fluid (ISF) from the epidermis and dermis by capillary action, reducing lag time and facilitating glucose monitoring in vivo. The prototype of our CGM device has extremely small size and demonstrated superior photo and signal stability for CGM in both small rodent and large animal models. Together, the collective findings in this work lay the essential groundwork for development of next generation CGM device based on fluorescent nanodiamond.

## 2 Results

### 2.1 Development of Boronic Hydrogel with Fluorescent Nanodiamond

A fluorescent glucose sensor with microneedle configuration can serve as a long‐term CGM device for diabetic patients without implantation (Figure 1A and Figure S1, Supporting Information). To ensure long‐term CGM in vivo, we need fluorophores with strong photostability and biocompatibility.^[^ ^19^, ^20^, ^21^ ^]^ Octadecylamine functionalized detonation nanodiamond has bright fluorescent signal and demonstrated anti‐inflammatory effects in vivo.^[^ ^22^, ^23^ ^]^ Octadecylamine functionalized nanodiamonds has also been used as a tracing material to monitor tissue regeneration in animal models.^[^ ^24^ ^]^ Embedding the nanodiamonds within a boronic polymer network can produce a dynamic sensor for fluorescence detection of glucose at physiological conditions. Changes in glucose concentration can alter density and hydration status of the boronic hydrogel, leading to changes in fluorescence transmission from nanodiamond (Figure 1B,C).

To prepare the octadecylamine modified nanodiamond, we used thionyl chloride to activate carboxyl group on the surface of the nanodiamond to generate acyl chloride and then reacted with amines to produce the amide (Figure S2A, Supporting Information). We further modified the nanodiamond using triethoxyvinylsilane to achieve alkene surface group for the covalent integration to the microneedle (Figure S2A, Supporting Information). The sequence of reaction was monitored by Fourier transform infrared spectroscopy to demonstrate unmodified nanodiamond, octadecylamine modified nanodiamond, and octadecylamine modified nanodiamond with alkene surface group (**Figure** **2**A). The weak peaks of the unmodified nanodiamond at 2912 cm^−1^ and the adjacent peak in the pristine nanodiamond can be attributed to the asymmetrical and symmetrical stretching vibration of hydrocarbon groups. The attachment of octadecylamine was verified by the dramatic increase of the intensity of hydrocarbon groups in modified nanodiamond, including the asymmetrical (2916 cm^−1^) and symmetrical (2850 cm^−1^) stretching vibrations, and the C–H scissor bending vibrations for methyl and methylene (1463 cm^−1^; 1373 cm^−1^). The amide bands at 1562 and 1640 cm^−1^ suggest covalent amide bond formation between octadecylamine and the nanodiamond. The strong Si–O–C stretching vibration bands at 1110 and 1020 cm^−1^ accompanied by a shoulder at 930 cm^−1^ are indicative for the grafting of triethoxyvinylsilane on modified nanodiamond. The functionalized nanodiamond particles have an average size of 2 ± 1 nm, as determined by transmission electron microscopy image (Figure S2B, Supporting Information) and dynamic light scattering (Figure S2C). The octadecylamine modified nanodiamond has maximum excitation at 370 nm and maximum emission at 450 nm in saline (Figure S2D,E, Supporting Information).

To integrate nanodiamond into the boric hydrogel, we prepared a semitransparent hydrogel by radical copolymerization of acrylamide, 3‐(acrylamido)‐phenylboronic acid, poly‐(ethylene glycol) diacrylate, and triethoxyvinylsilane surface modified fluorescent nanodiamond on a glass bottom of tissue culture dish (Figure S3A–C, Supporting Information). In the absence of glucose, the neutral phenylboronic acid groups are hydrophobic and tend to interact well between each other, resulting in a high optical density of microheterogeneric polymer network. In the presence of glucose, glucose molecules can reversibly form 1:1 complex with the phenylboronic acid derivatives. The Donnan osmotic pressure of the hydrogel will increase. In the meantime, the density, hydration status, and refractive index of hydrogel change, leading to enhanced light propagation efficiency through the hydrogel (Figure 1B,C). Consistent with this notion, confocal microscopy (excitation/emission wavelengths 400/450 nm) demonstrated differentially scattered nanodiamond particles in the hydrogel network at control (no glucose) or hyperglycaemic conditions (500 mg dL^−1^ of glucose) in vitro (Figure 2B,C), demonstrating the rearrangement of hydrogel structure upon glucose concentration changes. As expected, this rearrangement of the boronic polymer network leads to enhanced light transmission and fluorescent emission under hyperglycaemic condition, as observed with a fluorescence microscope (Figure 2D and Figure S4A, Supporting Information). Quantitative analysis shows a nearly linear correlation between fluorescent emission of nanodiamond and glucose concentration in vitro (Figure 2E). To examine the photostability of functionalized nanodiamond, we tested photobleaching in vitro in comparison with a classic glucose sensing hydrogel based on fluorescein isothiocyanate (FITC)–dextran and Concanavalin A (Con A). We found that exposure to excitation light (15 × 4 min, 6 W cm^−2^, 400 nm) can lead to 5–13% bleaching of fluorescein isothiocyanate‐dextran fluorescence in the hydrogel, whereas no appreciable photobleaching was observed for nanodiamond or nanodiamond based boronic hydrogel in vitro (Figure 2F and Figure S4B,C, Supporting Information). To assess the biocompatibility, we examined the potential cytotoxicity of our nanodiamond hydrogel. Our results show no significant changes of cell viability upon exposure to the hydrogel (Figure S4D, Supporting Information). Taken together, our results strongly suggest that the nanodiamond and boronic hydrogel material can serve as a safe and reliable biosensor for long‐term CGM.

### 2.2 Fabrication of Microneedles for Glucose Sensing

Eversense is an implantable fluorescence based CGM device. However, implantation and retrieval of the device from the patients have to be carried out surgically in a clinic setting. Fluorescence fibers have also been developed for examination of glucose concentration, but the application of the fibers is also invasive, and may lead to diminished patient compliance. To circumvent these issues, we aimed toward the development of a miniaturized skin‐wearable device with microneedle loading of nanodiamond hydrogel for CGM. However, the design and fabrication of a small but reliable device for optical detection of glucose are technically challenging.

**Figure 3:**
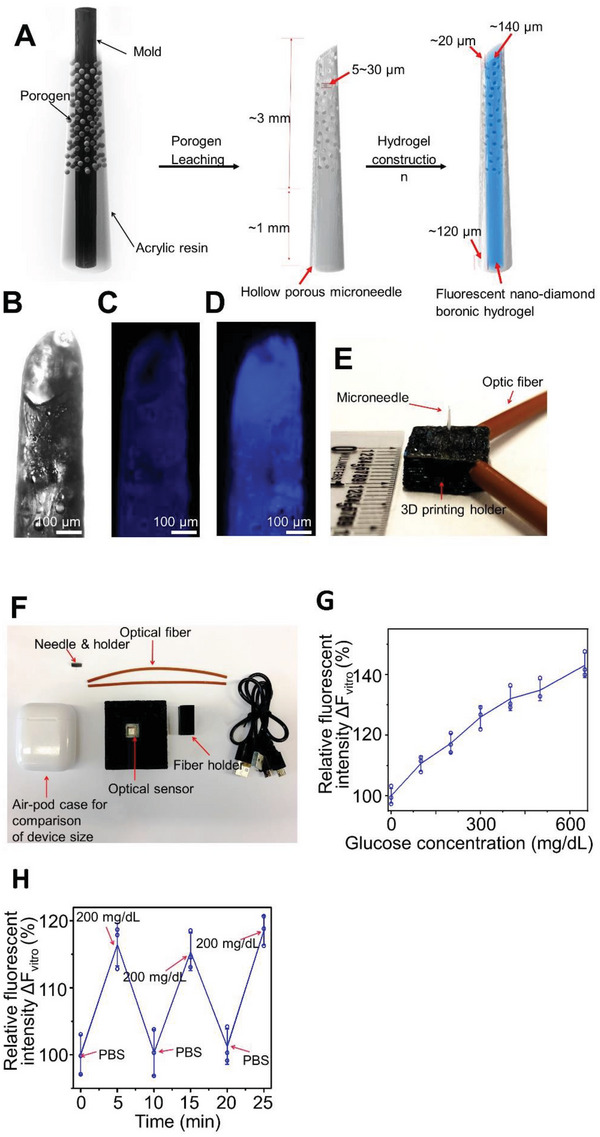
The device with fluorescent nanodiamond based boronic hydrogel can detect glucose in vitro. A) Schematic illustration of porogen leaching for the preparation of the porous microneedles and the structure of the microneedle. B) Microscopic image shows the porous structure of the microneedle. C,D) The background fluorescence of the porous microneedles was extremely low (C), whereas loading with nanodiamond based boronic hydrogel leads to significant fluorescent emission in vitro (D). E,F) Design and size comparison of the microneedle device with holder and optic fibers. G) The fluorescence intensity detected by the device correlates with glucose concentrations in vitro, ranging from 0 to 650 mg dL^−1^, *n* = 3. Data are presented as mean ± standard deviation (SD). All error bars represent SD. H) Changes of glucose concentration in vitro can be repeatedly measured by the microneedle device, *n* = 3. Data are presented as mean ± SD. All error bars represent SD.

To this end, we used porogen leaching technology to fabricate the microneedles with a porous hollow structure (**Figure** **3**A).^[^ ^25^, ^26^ ^]^ The nanodiamond based boronic hydrogel was then covalently constructed in the bore of the microneedle after silanization of the microneedle bore surface (Figure S3A,B, Supporting Information). The wall of the constructed microneedle exhibits a random open pore structure up to ≈3 mm from the sharp tip (Figure 3B–D and Figure S5A–D, Supporting Information). The blunt end of the microneedle (≈1 mm) was constructed without porogen, leading to an intact, uniform and transparent wall for an unfluctuating stable light transmission. The microneedle has a tip diameter of ≈180 µm, a base of ≈500 µm, and a length of ≈3 mm, which is significantly shorter than the needles used in the commercial CGM devices (≈10 mm). The wall thickness is ≈20 µm at the tip and ≈120 µm at the base. The pore size of the microneedle ranges from 5 to 30 µm. The fabricated microneedles have a tip angle of ≈40°. The porous microneedle design could enhance the diffusion of interstitial fluid into the needle while reducing the pain for device application.^[^ ^27^, ^28^, ^29^, ^30^ ^]^ Fluorescence imaging (excitation/emission wavelengths 400/450 nm) of the whole microneedle or cross‐section of the needle shows that the microneedle itself does not have significant autofluorescence, whereas embedded nanodiamond hydrogel exhibits strong fluorescence signals (Figure 3C,D and Figure S5B,C, Supporting Information).

### 2.3 Design and Fabrication of CGM device with Nanodiamond Hydrogel

A miniature and skin‐mountable device with the ability to transmit fluorescent signal from a minimally invasive microneedle has not been developed. To achieve these requirements, we used three‐dimensional (3D) printing approach to develop a light‐weight, rugged, and skin mountable device for glucose monitoring. Specifically, the device includes: (1) a rugged, miniature part to conjugate two optical fibers with a microneedle for excitation and transmission of the fluorescent signal from the nanodiamond hydrogel embedded inside the microneedles, and (2) a portable optic assembly consisting of a light‐emitting diode (emission wavelength 400 nm) as an incident light source and an optical sensor chip as a detecting module (Figure 3E,F and Figure S6A–C, Supporting Information). We tested the capability of glucose monitoring using our device assembled as described above in vitro. The fluorescence intensity at 450 nm was record with varying glucose concentrations (0–500 mg dL^−1^) to verify the monitoring capability within the normal (80–140 mg dL^−1^), hypoglycemic (<80 mg mL^−1^), and hyperglycemic (>140 mg dL^−1^) ranges.^[^ ^31^, ^32^ ^]^ When the glucose concentration increased from 0 to 650 mg dL^−1^, the fluorescence intensity collected by the device also increased (Figure 3G). The nanodiamond hydrogel responds to the glucose concentration changes in a reversible manner. The microneedle device maintains its sensitivity to glucose when it is subjected to repeated exposure to glucose‐free solution or glucose‐containing solution (200 mg dL^−1^) (Figure 3H). To determine the long‐term photostability of our system, we kept the loaded microneedles in phosphate‐buffered saline (PBS) buffer. Exposure to ambient light for up to 3 months does not compromise the capability of the microneedle device to sense the changes of glucose (Figure S7A, Supporting Information).

### 2.4 Detection of Glucose Concentration with Microneedle Device In Vivo

**Figure 4:**
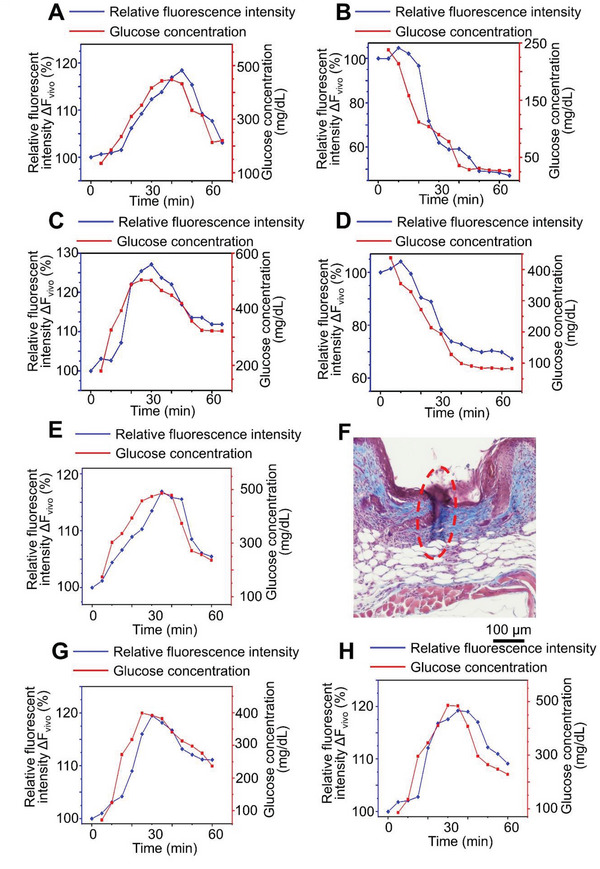
The microneedle device with fluorescent nanodiamond hydrogel can detect glucose concentration in vivo. A,B) Fluorescence intensity detected by the microneedle device continuously traced blood glucose level changes in glucose‐challenged (A) or insulin‐challenged (B) mice. C,D) Stability of the microneedle device. After 3 months of storage at ambient light, the device continues to detect glucose level in glucose‐challenged (C) or insulin‐challenged (D) mice. E) The sensor maintains good stability and response to glucose level change after 7 days of use on mouse skin. F) Trichrome staining demonstrates collagen deposition around the microneedles after 7 days of insertion. Red dashed lines denote lesion area. G,H) The sensor maintains reliable sensitivity to glucose level change after 21 (G) or 30 (H) days of use on mouse skin.

To test the glucose sensing capability in vivo, we mounted the microneedle device to mouse skin and carried out the IPGTT (intraperitoneal glucose tolerance test). Fasted animals were administered with a bolus of glucose intraperitoneally. The blood glucose level was determined by the nanodiamond hydrogel and a commercial glucose monitoring system (Bayer Contour) with blood samples taken from snipped tail simultaneously. **Figure** **4**A shows the correlation between the measured glucose concentration and the fluorescence level changes over time (Figure S7B, Supporting Information). Traditional glucose sensor cannot accurately measure low glucose level in vivo.^[^ ^2^, ^3^ ^]^ To test the microneedle sensor under lower glucose conditions, we induced hypoglycemia by insulin administration to fasted animals. The nanodiamond based device again demonstrated excellent correlation of the fluorescence signal with glucose level changes (Figure 4B and Figure S7C, Supporting Information). Consistent with our observation in vitro, exposure to ambient light for up to 3 months does not affect the sensitivity of our sensor in vivo. The microneedle device exhibited similar response to glucose level changes when mounted on mouse skin during IPGTT and under hypoglycemia condition (Figure 4C,D, and Figure S7D,E, Supporting Information). As our device detects glucose concentration in skin interstitial fluid, there is a lag time *t*_1/2_ of 3.8 ± 1.3 min during glucose increase, and 6.3 ± 3.3 min during glucose decrease (Figure 4A).^[^ ^33^ ^]^ Extended use of the sensor in vivo (7 days in mouse skin) does not significantly alter the lag time (*t*_1/2_ = 5.5 ± 1.3 min during glucose increase, and *t*_1/2_ = 8.5 ± 3.4 min during glucose decrease). Tissue fibrosis or long‐term storage of the sensor (up to 3 months) does not change the lag time either (Figure 4C–F). We further conducted long‐term tests of our device on mouse skin. Our device exhibits reliable sensitivity for glucose sensing in vivo for 30 days (Figure 4G,H). Conventional CGM devices with electrochemical approach for glucose sensing can be significantly affected by magnetic field interference, deoxygenation, and temperature changes. In comparison with the commercial product, FreeStyle Libre 2, our sensors demonstrated superior stability and reliability under these conditions (Figures S8–S10, Supporting Information).

**Figure 5:**
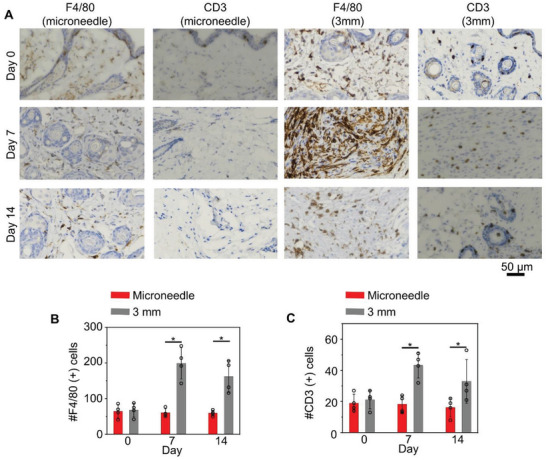
Microneedle insertion is minimally invasive to skin. A) Sections of the skin after microneedle insertion or full‐thickness skin wounds (3 mm) were stained with F4/80 or CD3 antibodies to visualize skin immune infiltration. B) Quantification of F4/80 and CD3 positive cells in skin samples, *n* = 4. Data are presented as mean ± standard deviation (SD). All error bars represent SD. *p < 0.05 (*t*‐test).

Insertion of the microneedle leaves a small wound (≈300 µm) in the murine skin. Only mild and localized erythema was observed immediately after needle application, which dissolved within minutes. To assess potential changes in inflammatory responses upon microneedle application, we collected and stained skin tissue sections with F4/80 for macrophages, and CD3 for T lymphocytes. Compared to full‐thickness skin wounds (3 mm), application of microneedles leads to significantly less immune infiltration after glucose monitoring experiment (**Figure** **5**A,B, and Figure S11, Supporting Information). Together, our results strongly suggest that the microneedle device is a safe and minimally invasive approach for long‐term monitoring of glucose level in vivo.

### 2.5 Validation of the Microneedle Device in Large Animals

**Figure 6:**
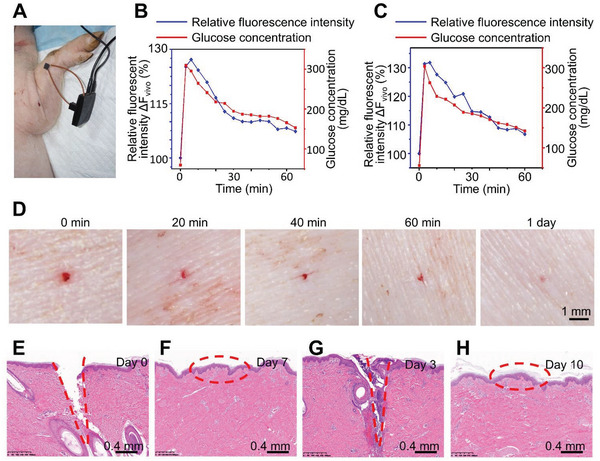
The microneedle device with nanodiamond hydrogel can detect glucose changes in large animal models. A) Experimental setup for the device to detect blood glucose level in vivo with pig model. B,C) Fluorescence intensity detected by the device continuously traced blood glucose level change in glucose‐challenged pig from day 0 (B) to day 3 (C). D) Photograph of porcine skin after glucose monitoring experiment and microneedle retrieval. E,F) Representative hematoxylin and eosin staining of porcine skin at day 0 (E) and day 7 (F) after microneedle removal. G,H) After continuously monitoring of blood glucose level for 3 days, representative hematoxylin and eosin staining revealed defined lesions in the epidermis and dermis at day 3 (G). Skin wounds were healed after 7 days (H). Red dashed lines denote lesion area.

Porcine skin resembles human skin anatomically and physiologically.^[^ ^34^ ^]^ To examine the performance of the microneedle device in a large animal model, we performed blood glucose level monitoring in pigs (**Figure** **6**A). With similar IPGTT paradigm, we found that the fluorescence intensity recorded by our device effectively tracks the blood glucose level fluctuation in vivo and correlate with the readings from commercial glucose meter (Figure 6B and Figure S12, Supporting Information). We then performed CGM with freely moving animals for 3 days. Our device successfully monitored the glucose concentration and traced the actual blood glucose levels of jugular vein samples measured every few minutes by commercial glucometer (Figure 6C). The microneedle device is minimally invasive to porcine skin. Figure 6D shows the sequential images of pig skin wounds after removal of the microneedle monitoring device. Histological examination of porcine skin revealed defined lesions in the epidermis and dermis immediately after needle insertion (Figure 6E), and complete wound healing 7 days after removal (Figure 6F). Three days CGM with our device on free moving pigs showed no significant skin inflammation (Figure 6G) and the skin also totally recovered 7 days after removing the device (Figure 5H). This kinetic is similar to observations made in human subjects.^[^ ^35^ ^]^

## 3 Discussion

A biointegrated sensor for noninvasive monitoring of blood glucose level in vivo will remove the need for patients to draw blood multiple times a day.^[^ ^2^, ^3^, ^5^ ^]^ Additionally, continuous monitoring of glucose allows the patients to better maintain their blood glucose level by altering their insulin dosage or diet according to the prevailing glucose values and prevents potential hyperglycemia or hypoglycemia.^[^ ^36^, ^37^, ^38^ ^]^ Continuous monitoring of glucose can be also used for screening gestational diabetes mellitus which affects approximately 6% of pregnancies in the United States.^[^ ^39^ ^]^ If the sensor can be connected to an insulin delivery device, it may create an “artificial endocrine pancreas” that could automatically maintain the glucose level in patients. Currently, most of the continuous monitoring sensors for glucose are enzyme electrodes or microdialysis probes implanted under the skin.^[^ ^2^, ^3^, ^5^ ^]^ These sensors usually require oxygen for activity, are insufficiently stable in vivo, and exhibit poor accuracy under low glucose conditions. The presence of interfering electroactive substances in tissues can also cause impaired responses and signal drift in vivo, which necessitates frequent calibrations of current sensors. A fluorescence‐based glucose sensor in the skin will likely be more stable, have improved sensitivity, and resolve the issue of electrochemical interference from the tissue.^[^ ^4^, ^40^, ^41^, ^42^, ^43^, ^44^ ^]^ In this study, we developed a nanodiamond based glucose sensor.

Most CGM detect the glucose level in the body fluids, including interstitial fluid, sweat, and tears.^[^ ^45^, ^46^ ^]^ Because an adequate level of hydration is essential for the diffusion of glucose molecules, all CGMs, including our system, require sufficient hydration.^[^ ^38^, ^47^ ^]^ However, unlike other enzyme based electrochemical CGMs that can be affected by the extensive parasitic capacitive coupling, our optical system does not require electrode and thus electrolyte concentration does not significantly influence the system's response to glucose.^[^ ^40^ ^]^ In direct comparison with FreeStyle Libre 2, our sensors are not affected by environmental magnetic field, temperature changes, or buffer deoxygenation. Other polyols from interstitial fluid, such as D‐fructose can also bind to boronic acids. But, concentration of other polyols in interstitial fluid is much lower compared with D‐glucose, so it will unlikely affect the reading of our sensor (D‐glucose 4–7 × 10^−3^ m; D‐fructose <0.1 × 10^−3^ m).^[^ ^48^ ^]^ Our proof‐of‐concept study shows that a microneedle device with this material can serve as noninvasive and long‐lasting fluorescence sensor for glucose in vivo. Optical sensors for glucose have been tested before, such as Concanavalin A or polymer‐dot labeled glucose oxidase.^[^ ^49^, ^50^ ^]^ However, fluorescently labeled concanavalin A is not a safe/biocompatible material for long‐term CGM because it has been associated with a variety of toxicities in vivo, such as mitogenic, cytotoxic, hepatotoxic, and teratogenic toxicities.^[^ ^51^ ^]^ For polymer dot labeled glucose oxidase, the system relies on enzymatic reaction, which can be affected by biodegradation, oxygen level, temperature, and foreign body reactions.^[^ ^50^, ^52^, ^53^ ^]^ Although the current Eversense optical sensor (Senseonic) can be used for 6 months, the device is bulky (18.3 × 3.5 mm) and has to be implanted under the patient skin through surgical approach, which limits its applicability and reduces patients’ compliance. A recent study has demonstrated many adverse effects upon implantation of the device, including bruising, erythema, or pain/discomfort from a 90‐day data collection.^[^ ^54^ ^]^ In addition, as other CGM devices, Eversense sensor cannot function properly under high magnetic/electrical field because of the core electronics inside. For patients who need magnetic resonance imaging (MRI) or computed tomography (CT) scan, removal of the implanted sensor is time consuming but leaving the sensor inside can be dangerous. Furthermore, the long‐term implantation of a large glucose sensor may lead to inflammatory response and tissue fibrosis. By contrast, our system is based on microneedle and novel diamond‐hydrogel. Our sensor is small in size, minimally invasive, and easy to apply/remove, providing a safer and more user friendly optical CGM device for diabetes patients.

Stimuli‐responsive polymer conjugation has been used to enhance responsiveness of nanomaterial toward temperature, potential of hydrogen (pH), redox environment ionic strengths, light, electric/magnetic fields, or biomolecules changes.^[^ ^55^, ^56^, ^57^, ^58^ ^]^ Among the many natural and synthetic materials employed as molecular sensors, boronic acids have been found to be particularly useful for glucose sensing as they can fast and reversibly bind to glucose by covalent boronate formation.^[^ ^59^ ^]^ Most studies including currently marketed CGM products used organic fluorescent derivatives coupled with boronic acid polymer for glucose sensing. However, conventional organic fluorophores can be unstable under physiological conditions, easily colored, have poor photostability.^[^ ^37^, ^44^, ^60^, ^61^, ^62^, ^63^, ^64^, ^65^, ^66^ ^]^ The organic fluorophores may also increase the reactive oxygen species in vivo and exhibit dose independent cytotoxicity.^[^ ^57^ ^]^ Although inorganic fluorophores such as quantum dots (cadmium telluride or cadmium selenide) have demonstrated their unique optical properties including brightness, stability, and size‐dependent fluorescence, the intrinsic toxicity of heavy metals and chalcogenides limits their applications in vivo.^[^ ^67^, ^68^, ^69^, ^70^, ^71^, ^72^ ^]^ Different from the conventional quantum dots, fluorescent nanodiamond has superior biocompatibility. In addition, fluorescent nanodiamond is easy for large‐scale synthesis and inexpensive. Fluorescent nanodiamond also has facile surface for bioconjugation and strong photostability,^[^ ^73^, ^74^, ^75^, ^76^, ^77^, ^78^ ^]^ making it an ideal candidate for long‐term CGM in vivo. In this study, we employed a free radical copolymerization approach to covalently functionalize fluorescence nanodiamond with phenylboronic acid for glucose sensing. Our results demonstrated outstanding glucose‐responsiveness, stability, and biocompatibility of nanodiamond based CGM material in small and large animal models in vivo.

Biointegrated sensors can address various challenges in medicine by transmitting a wide variety of biological signals, including electrophysiological and biochemical signals continuously.^[^ ^79^ ^]^ Recent advancement in device designs and fabrication using novel nanomaterials have greatly accelerated the development of new soft and stretchable electronics applicable in vivo, including brain, heart, and skin.^[^ ^79^, ^80^ ^]^ Interstitial fluid is an extracellular liquid that accumulates inside the tissue and is implicated in the regulation of the local tissue environment, serving as a reservoir of biomolecules, nutrients, and metabolic products.^[^ ^81^, ^82^ ^]^ Although many new technologies for detecting the biomarkers in interstitial fluid have been proposed, most of them require enzymatic reaction or other special sample processing before testing, which greatly limits their applications in vivo.^[^ ^83^, ^84^, ^85^ ^]^ In this regard, our optically responsive hydrogel in microneedle system can potentially translate different biochemical signals into fluorescence changes, making it an attractive platform for noninvasive biomedical monitoring system. For instance, cell‐free nucleic acids in interstitial fluid have been reported as predictive, diagnostic, and prognostic biomarkers for cancers.^[^ ^86^, ^87^, ^88^ ^]^ The nanodiamond hydrogel can be functionalized with peptide nucleic acid capture probes for sequence specific immobilization of the target nucleic acids via Watson−Crick base pairing, and thus detect the corresponding nucleic acids through similar optical response.^[^ ^89^ ^]^ Level of inflammatory cytokines such as tumor necrosis factor‐alpha, interleukin (IL)‐1*β*, and IL‐6 can change in interstitial fluid under different pathophysiological conditions,^[^ ^84^, ^90^, ^91^, ^92^ ^]^ which can be detected by the fluorescent nanodiamond hydrogel by incorporation of different antibodies against the cytokines. By integrating the hydrogel in microneedle with pH responsive units such as pH‐responsive solvatochromic dye, polyaniline, or poly(*N*‐isopropylacrylamide), our device can also be used for in situ detection of pH, which can be useful for diagnostic of epilepsy.^[^ ^93^, ^94^, ^95^ ^]^

Taken together, our study demonstrates that a microneedle device with functionalized nanodiamond hydrogel network can be used to accurately monitor glucose changes in vivo. Our study unravels the tempting potential of skin mountable device for various clinical applications in the future.

## 4 Experimental Section

### Materials

Nanodiamond (ND50 grade) was purchased from Dynalene, Inc., USA. Thionyl chloride, hydrogen chloride (HCl), *N*,*N*‐dimethylformamide, tetrahydrofuran, octadecylamine, methanol, triethoxyvinylsilane, sodium chloride, glucose, ethanol were purchased from Sigma‐Aldrich. Acrylic plastic casting resin and hardener were purchased from Electron Microscopy Sciences. Optical fiber (UV/VIS, 400 µm diameter) was purchased from Vernier. A 5‐mm light‐emitting diode (400 nm, DC 12 V), 560 Ω ohms 1/4 W resistor, and 24 awg wire were purchased from Chanzon. Spectral sensor (9 × 9 mm, 8 band, 425–775 nm) and electronics board (45.72 × 21.34 mm, OEM) were purchased from Pixelteq. Polylactic acid filament was purchased from Overture. Fine test sieve was purchased from Sigma‐Aldrich. Recombinant human insulin was purchased from Sigma‐Aldrich. NIH/3T3 cells lines were purchased from ATCC. All other analytical grade reagents were purchased from Sigma‐Aldrich.

### General Methods

Transmission electron microscopy (Tecnai Spirit TEM), FTIR spectrometer (Nicolet iS50), lab water system (Millipore Milli‐Q), muffle furnace (Nabertherm), confocal microscope (Leica SP5), blue light lamp (Amsuns, 460 nm), dynamic/electrophoretic light scattering (Wyatt Möbiu*ζ*), microplate reader (BioTek Synergy neo), inverted microscope (Nikon ECLIPSE Ti2), lab centrifuge (Sorvall Legend Micro 21), vortexer mixer (VWR, mini vortexer), digital microscope (T Takmly, MX200‐B), scope filter (bandpass 450 nm, Optical Filter Shop), and 3D printer (X‐Plus, R Qidi Technology) were employed for materials preparation and characterization.

### Preparation of Fluorescent Nanodiamond

Fluorescent nanodiamond was prepared according to procedures inspired from the literatures and as described previously.^[^ ^57^, ^75^, ^96^ ^]^ First, 30 mg nanodiamond powder was heated at 425 °C for 5 h in a furnace to remove the surface graphite, and cleansed of metal impurities by boiling in 35 wt% HCl for 24. The particles were then refluxed with 50 mL of thionyl chloride and 1 mL of anhydrous *N*,*N*‐dimethylformamide at 70 °C for 24 h. The brown liquid was removed and the nanoparticles were washed two times with anhydrous tetrahydrofuran. After dried under vacuum at ambient temperature, the acylchloride derivative nanodiamond was stirred in a sealed flask with 1 g of octadecylamine at 100 °C for 4 days. After ultrasonicated and washed with anhydrous methanol five times to remove excess octadecylamine, the solid was reflux with 2 mL of 10 m sodium hydroxide for 3 h. The nanodiamond was washed three times with Milli‐Q water, and then stirred with 0.03 mL triethoxyvinylsilane, 1.9 mL ethanol, and 0.07 mL Milli‐Q water for 3 h. At last, the synthesized blue fluorescence nanodiamond with vinyl groups on the surface was collected after washed with ethanol three times and vacuum dried at ambient temperature.

### Preparation and Characterization of Fluorescent Nanodiamond Based Boronic Hydrogel

The glass bottom of a 35 mm tissue culture dish (FluoroDish, World Precision Instruments, Inc.) was first treated with triethoxyvinylsilane, ethanol, and water (3: 190: 7 vol%) solution overnight. The fluorescent nanodiamond based boronic hydrogel prepared according to procedures inspired from the literatures.^[^ ^97^ ^]^ 156 mg of acrylamide, 76.4 mg 3‐(acrylamido)phenylboronic acid, and 56 mg poly(ethylene glycol) diacrylate (Mn 700) were dissolved in 0.1 wt% blue fluorescence nanodiamond water suspension by ultrasonication. 5 µL *N*,*N*,*N*’,*N*’‐tetramethylethylenediamine was added to the suspension and then degassed. Immediately after mixing 2 µL of the suspension with 1 µL of 40 mg mL^−1^ fresh ammonium persulphate solution on the glass bottom of 35 mm tissue culture dish, the hydrogel was formed on the cover glass after approximate 10 s. The fluorescent nanodiamond based hydrogel was dialyzed in 1× sodium phosphate buffer solution overnight to remove the unreacted molecules. After that, it was stored in 1× sodium phosphate buffer solution.

As showed in Figure S4A (Supporting Information), the fluorescent nanodiamond based boronic hydrogel was placed under 3 mL of different concentration glucose solutions. An optical fiber casted a 400 nm exaction light from 45° above. A digital microscope with a scope filter (bandpass 450 nm) was placed under the hydrogel. The fluorescence intensity was estimated as described in previous literatures by analyzing the obtained fluorescent images using image processing software (ImageJ).^[^ ^43^, ^98^ ^]^

### Cytotoxicity of Fluorescent Nanodiamond Based Boronic Hydrogel

The cytotoxicity was assessed by the WST‐1 method inspired from previous literatures.^[^ ^63^, ^99^, ^100^ ^]^ NIH/3T3 cells were grown in ATCC‐formulated Dulbecco's Modified Eagle's Medium (Catalog No. 30–2002, 10% bovine calf serum). Cells were incubated at 37 °C in an atmosphere of 5% carbon dioxide and 95% air and underwent passage twice a week. The cells were maintained according to routine cell culture procedures. To determine cell viability after exposure of all cell lines to different concentrations incubated for 24 h, the CCK‐8 assay was performed according to manufacturer's instructions. Briefly, cells were seeded in a 24 wells plate at 50 000 cells per well. After incubated in humidified atmosphere (90% humidity), 7.5% CO_2_ at 37 °C for 24 h, the fluorescent nanodiamond based boronic hydrogel (as prepared above and carefully peeled from the glass bottom of the tissue culture dish) was placed in strainers and sunk into cell growth medium. After incubating for 1 and 3 days, the medium was removed, and after washing the cells with PBS, cell was incubated with 15 µL of WST‐1 reagent per well for 1 h, followed by analyzing the measurement of the absorbance at 440 nm with plate reader (the well with reagent and no cell served as the blank control).

### Comparison of Photostability

The Con A hydrogel was prepared according to a previous literature.^[^ ^101^ ^]^ Briefly, Con A (10 mg mL) and FITC‐Dextran (0.1 mg mL) were mixed in 40:1 mass ratio and incubated for 30 min to complete complex formation. Molten agarose (4%, low‐melting) was mixed with the complex solution in 1:1 (v/v) ratio at ≈37 °C. Three microliters of the mixture was dropped onto the center of well (96 wells glass bottom black plate). Hydrogel pads with diameters of ≈1.5 mm were formed and allowed to stand in dark room for 30 min at room temperature for solidification. Two microliters of 0.1 wt% blue fluorescence nanodiamond water suspension was added to another well. Four replicas were made for each analyte and 100 µL of 100 mg dL glucose in PBS solution was added into each well. After a set time of blue light exposure (15 × 4 min, 5 mW cm^−2^, 460 nm; Light resource: Coast PX100. Condition: 15 × 4 min, 6 W cm^−2^, 400 nm), the fluorescence was measured with a plate reader.

### Fabrication of Porous Microneedle

The porous microneedle was prepared according to procedures inspired from the literatures.^[^ ^102^, ^103^ ^]^ The salt leaching method was employed to realize the porous structure of the microneedle. The acrylic resin was mixed with salt, followed by washing out the salt with water, which resulted in the intended pores inside the microneedle. The pore size was controlled by employing a fine test sieve with pore size 38 µm to get sodium chloride particles with size ≤40 µm, which was small enough for an efficient ISF collection and provided a good connection of the obtained porous structure to the hydrogel inside the microneedle.^[^ ^103^ ^]^ In this study, acrylic resin (resin: hardener, 500:1, volume ratio) with was mixed with salt at a volume ratio of 7%. Acrylic resin and salt were coarsely mixed by a manual operation, followed by mixing using a vortexer mixer to achieve a uniform distribution of salt. A 10 µL pipette tip (Dot Scientific) and a syringe needle (30 G, BD) were used as mold. The salt‐mixed resin was cast into the mold till around 4 mm high manually with a syringe (1 mL, BD). After that, an acrylic resin (resin: hardener, 500: 1, volume ratio) without porogen was also cast into the mold till around 5 mm high. After 24 h of hardening, the mold was removed carefully and the hollow resin needle was exposed to water for 48 h to dissolve the salt. The tip of the microneedle was sharpened by a sanding wheel (Dremel, 686‐01) to make a tip angle around 40°.

### Construction of Fluorescent Nanodiamond Based Boronic Hydrogel in the Porous Microneedle

The surface of the porous microneedle was first silanizated according to procedures inspired from the previous literature.^[^ ^97^, ^104^ ^]^ The microneedle was extensively washed with ethanol and deionized water to remove any surface impurities. The microneedle samples were treated in 2 m hydrochloric acid solution for 3 h followed by a wash in Milli‐Q water for 5 min. The microneedle was then treated with triethoxyvinylsilane, ethanol, and water (3:190:7 vol%) solution overnight. The polymer part of glucose responsive hydrogel was synthesized as a phenylboronic acid functionalized polyacrylamide gel. An amount of 156 mg of acrylamide, 76.4 mg 3‐(acrylamido)phenylboronic acid, and 56 mg poly(ethylene glycol) diacrylate were dissolved in 0.1 wt% blue fluorescence nanodiamond water suspension by ultrasonication. A total of 5 µL *N*,*N*,*N*’,*N*’‐tetramethylethylenediamine was added to the suspension and then degassed. Immediately after mixing 200 µL of the suspension with 100 µL of 40 mg mL^−1^ fresh ammonium persulphate solution, the microneedle was placed in the mixture under vacuum for 10 s. The microneedle was then taken out and let the hydrogel formed in the microneedle. The microneedle with fluorescent nanodiamond based hydrogel was dialyzed in 1× sodium phosphate buffer solution overnight to remove the unreacted molecules. After that, it was stored in 1× sodium phosphate buffer solution.

### Device Configurations

The microneedle holder was made using 3D printing. Polylactic acid was used as filament materials. The dimensions are illustrated in Figure S6A (Supporting Information). The width, length and height of the holder were 6, 10, and 2 mm. The depth and width of the groove were 0.5 mm. The included angle of the two optical fibers was 135°. After conjugation of the microneedle with the two optical fibers from the above mentioned device, the groove was sealed and the microneedle was fixed with hot melt glue (Gliston). As shown in Figure S6 (Supporting Information), the all‐in‐one multifunctional chip was composed with an optical sensor (includes a spectral sensor and an electronic board), and a wired light‐emitting diode with resistor in a 50 × 40 × 7 mm black box (customized made by 3D printing to separate all units from influence each other). One of the optical fibers (length 10 cm) linked to the microneedle was conjugated to the light‐emitting diode by heat shrink tube (Ginsco), while the other optical fiber was placed on top of the spectral and further fixed with a fiber holder (1 × 1 × 2 cm, customized made by 3D printing).

### Measurement of Glucose Concentration In Vitro

The as fabricated microneedle was dipped into 2 mL of PBS buffer with different glucose concentration in a Petri dish at room temperature. After 5 min of gently pipetting up and down the solution with a 1 mL pipette for better glucose diffusion, the emission intensity was collected from the spectral sensor software (Pixelteq Eval GUI). The liquid was sucked away from the dish using syringe after the collection of data. Then 2 mL of a fresh solution was inserted to the Petri dish again and the emission intensity was collected again. These operations were repeated several times. Fluorescence intensity for the glucose concentrations of 0–500 mg dL^−1^ was first measured. Next, the reversible coordination of emission intensity to glucose concentration was tested. The buffer was switched three times with glucose concentration from 0 to 200 mg dL^−1^.

For photostability test, the fluorescent nanodiamond based boronic hydrogel was first prepared in the porous microneedle as described above. Then, it was kept in a clear microcentrifuge tube (Eppendorf, 1.5 mL, made of polypropylene) filled with PBS buffer under ambient light for 3 months. The device was assembled and the reversible coordination of emission intensity to glucose concentration in vitro was tested again using the same method.

All signals were averaged five times and three measurements were taken for each time point. The relative fluorescence intensity Δ*F*_vitro_ was estimated according to the below equation.

(1) $$\Delta F_{\mathrm{vitro}}=100\%\times F÷F_{\mathrm{PBS}}$$

F: Fluorescence intensity of the tested buffer
F_PBS_: Mean fluorescence intensity of the PBS buffer.

### In Vivo Blood Glucose Level Monitoring

The in vivo blood glucose level monitoring with the fluorescent nanodiamond based device was performed using nine male mice (C57BL/6J, the Jackson Laboratory, 9‐week‐old), weighting 21–16 g, and two male hybrid pig, weighing 21 and 23 kg, respectively. All mice used in this study were bred and maintained at the ARC (animal resource center) of the University of Chicago in accordance with institutional guidelines. All the experimental procedures on live animals (mouse) were carried out in line with the Institutional Animal Care and Use Committee (IACUC) approved protocols of the Animal Care Center at the University of Chicago. All pig experimental procedures were approved by the Ethics Committee of Changhai Hospital, Shanghai, China. All the mice were housed under pathogen‐free conditions in the ARC (Animal Resources Center) at the University of Chicago under a 12 h light–dark cycle. Housing facility maintains a temperature at 70–73 °F (average 72) and humidity at 40–50% (average 44%). All pigs were housed under the temperature 24 ± 2 °C and relative humidity 30–70%, and given free access to food and water with a 12 h light–dark cycle. All the subjects were not involved in any previous procedures. Microneedle was sterilized prior to surgery using a 20 µL drop of 70% ethanol.

Before test, mice were anesthetized using intraperitoneal injection with 10 µL g^−1^ body weight drug cocktail (1 mL of Ketamine HCL (100 mg mL^−1^, Hospira), 0.8 mL of xylazine (20 mg mL^−1^, Akorn), and 8.2 mL of sterile water), and had their test site on dorsal shaved and sterilized with 70% ethanol.

Tests were performed on four mice with 1–3 glucose challenges or insulin challenges individually. For glucose challenge, mice were fasted for 8–10 h before the experiment. The emission light intensity from the device was recorded 5 min after the microneedle was inserted into the dorsal skin of mouse. Then, 10 wt% glucose solution (1.5 g kg^−1^ glucose/body weight) was injected into mice through intraperitoneal injection. Five minutes after that, blood glucose concentrations were measured using a standard glucose meter (EZ meter; Bayer Contour NEXT) by blood sample from the snipped tail every 5 min. The emission light intensity from the device was also recorded every 5 min. For insulin challenge, mice were fasted for 8–10 h before the experiment. Then the mice were injected with 10 wt% glucose solution (1.5 g kg^−1^ glucose/body weight) through intraperitoneal injection. After 20 min, the microneedle was inserted into the dorsal skin of mouse. The emission light intensity from the device was recorded after 5 min. Then, recombinant human insulin (2 Unit kg^−1^ glucose/body weight) was injected into mouse through intraperitoneal injection. Five minutes after that, blood glucose concentrations were measured using a standard glucose meter by blood sample from the snipped tail every 5 min. The emission light intensity from the device was also recorded every 5 min. For long‐term blood glucose level monitoring test, the microneedle was left on the dorsal skin for 7 days. After that, the glucose challenge experiment was performed as above described.

For additional photostability test, the fluorescent nanodiamond based boronic hydrogel was first prepared in the porous microneedle as described above. Then, it is kept in a clear microcentrifuge tube (Eppendorf, 1.5 mL, made of polypropylene) filled with PBS buffer under ambient light for 3 months. The device was assembled and the glucose challenges or insulin challenges were tested on four mice again using the same method as described above.

The pigs were anesthetized with an injection of ketamine (20 mg kg^−1^, IM) and maintained with propofol (4 mg kg^−1^ h^−1^). For stress‐free and frequent blood sampling, central venous catheters were inserted into the external jugular vein. Pigs were fasted for 15 h before the experiment. The emission light intensity from the device was recorded 5 min after the microneedle was inserted into the skin of front leg. After that, a bolus injection of 50% glucose solution was administered at 0.5 g kg^−1^ of body weight. The emission light intensity from the device was recorded at 0, 3, 6, 10, 15, 20, 25, 30, 35, 40, 45, 50, 55, 60, and 65 min after the microneedle was inserted into the dorsal skin of mouse. Blood was collected at the indicated time points and blood glucose levels were measured with Cofoe glucometer (Hunan, China).

For long‐term in vivo blood glucose level monitoring test, the microneedle was left inserted in the skin of front leg for 72 h. After that, the experiment was performed as above described. The emission light intensity from the device was recorded at 0, 3, 6, 10, 15, 20, 25, 30, 35, 40, 45, 50, 55, and 60 min after the injection of 50% glucose solution. Blood was also collected at the indicated time points and blood glucose levels were measured with Cofoe glucometer (Hunan, China).

All signals of the fluorescence intensity were averaged five times for each time point. The relative fluorescence intensity Δ*F*_vivo_ was estimated according to the below equation:

(2) $$\Delta F_{\mathrm{vivo}}=100\%\times F÷F_{0}$$

*F*: Fluorescence intensity of the tested mouse
*F*_0_: First fluorescence intensity of the tested mouse before glucose or insulin injection

### Histopathology

After the experiments, the experimented mouse or pig skin was cut off. The targeted area was the incised skin. Skin tissues were embedded in the Tissue‐Tek optimal cutting temperature compound and cryosectioned into 5‐µm slices. The tissue slabs were processed by standard histological procedures, histochemically stained with hematoxylin and eosin (H&E), F4/80 antibody, trichrome, and CD^3+^ antibody.^[^ ^105^, ^106^ ^]^ Antibodies were diluted according to manufacturer's instruction, unless indicated otherwise. Serving as a control, two wounds were created on the dorsal side of each mouse by removing full‐thickness skin via 3‐mm punch biopsy. Tegaderm films (3 M Inc.) were used to cover the wounds and prevent water loss in all the mice until the wounds were fully epithelialized. At days 7 and 14 postsurgery, three mice in each group were euthanized and the wounded skin removed, fixed in formalin, embedded in paraffin, and sectioned. Trichrome, F4/80 antibody, and CD^3+^ antibody staining were used for histological observations. Microscopic evaluation of the tissue sections was performed after that. The sections with F4/80, and CD^3+^ staining were observed under the microscope (Eclipse Ti2; Nikon Inc.) at 20× and 200× magnification. Four fields were randomly selected from each section to count the F4/80 positive macrophages, and CD^3+^ positive T‐cells. Immunoreactive cells were quantified as the mean cell count expressing the appropriate positive marker per high‐power field (HPF). Histological data were expressed as mean ± standard deviation (SD). Statistical analysis was performed by Student's *t*‐test. A *p* value of < 0.05 was considered significant.

### Statistical Analyses

Comparisons between quantitative data were conducted using the unpaired or paired Student's *t*‐test, Mann–Whitney *U*‐test, or Dunnett's *t*‐test, where appropriate. All *p* values were two‐tailed and *p* values of 0.05 or less were considered to be statistically significant (*p < 0.05, **p < 0.01, ***p < 0.001).

## Conflict of Interest

The authors declare no conflict of interest.

## Author Contributions

J.Z., Y.Z., and J.L. contributed equally to this work. X.W., Q.L., C.Y., and J.Z. designed the experiments. J.Z., Y.Z., J.L., S.A.K., L.C., and J.Z. performed the experiments. J.Z., Y.Z., J.L., L.Z., and X.W. analyzed the data. J.Z. and X.W. wrote the manuscript. All authors edited the manuscript.

## Supporting information

Supporting Information

## Data Availability

The data that support the findings of this study are available from the corresponding author upon reasonable request.
