# Supplementary material for: Continuous Glucose Monitoring Enabled by Fluorescent Nanodiamond Boronic Hydrogel
Source: Adv Sci (Weinh). 2023 Jan 16;10(7):2203943. doi: 10.1002/advs.202203943 (PMC9982560; doi:10.1002/advs.202203943)
Supplement: Supplementary file 1 — Supporting Information [file ADVS-10-2203943-s001.pdf]

SUPPLEMENTARY INFORMATION

Supplementary Table 1

List of Abbreviation

Abbreviation	Definition
CGM	Continuous glucose monitoring
FND	Fluorescent nano-diamond
FRET	Fluorescence resonance energy transfer
FDA	The Food and Drug Administration
ISF	Interstitial fluid
PBS	Phosphate-buffered saline
pH	Potential of hydrogen
FITC	Fluorescein isothiocyanate
Con A	Concanavalin A
3 D	Three-dimensional
IPGTT	Intraperitoneal glucose tolerance test
IL	Interleukin
HCl	Hydrogen chloride
MRI	Magnetic resonance imaging
CT	Computed Tomography
UV/VIS	Ultraviolet/Visible
FTIR	Fourier transform infrared
ARC	Animal resource center
IACUC	The Institutional Animal Care and Use Committee
IM	Intramuscular
H&E	Hematoxylin and Eosin
HPE	High-power field
SD	Standard deviation

Figure S1: Schematic illustration of the skin-mountable device for fluorescent detection of glucose.

Figure S2: Fabrication of fluorescent nano-diamond hydrogel for glucose sensing. (A) Surface functionalization of the nano-diamond with octadecylamine and triethoxyvinylsilane. **(B-E)** Fluorescent nano-diamond was characterized by transmission electron microscopy image (B), dynamic light scattering (C), emission spectra at 355, 370, 380, 400, and 410 nm excitation (D), and excitation spectra at 400 and 450 nm emission (E).

Figure S3: The preparation of fluorescent nano-diamond based hydrogel. (A) The chemical equation of silanization of the bore of the microneedle. **(B)** The glucose responsive hydrogel was synthesized as a polyacrylamide gel with fluorescent nano-diamond and phenylboronic acid functionalization. **(C)** Photograph of the fluorescent nano-diamond based boronic hydrogel prepared on the glass bottom of a tissue culture dish.

Figure S4: Characterization of the fluorescent nano-diamond based boronic hydrogel. (A) Experimental setup to detect fluorescence emission from the nano-diamond based boronic hydrogels in glucose solution. **(B)** Nano-diamond based hydrogel exhibited excellent photostability with 15 × 4 minutes, 5 W/cm^2, 460 nm exposure. n = 4. Data are presented as mean ± SD. All error bars represent SD. **(C)** Value of the relative fluorescent intensity over 4000 cyclic illumination cycles for the control and the nano-diamond based hydrogel. 1 illumination cycle represents 1 second of illumination using a LED with a wavelength of 400 nm and a power of 6 W/cm^2. **(D)** No significant cytotoxicity of the nano-diamond hydrogel was found on day 1 and day 3 with the WST-1 cellular viability assay. n = 4. Data are presented as mean ± SD. All error bars represent SD.

Figure S5: Characterization of the microneedle for glucose sensing. (A) Microscopic image of the microneedle bottom. **(B-C)** Fluorescence images of the microneedle bottom without (B) or with (C) nano-diamond based hydrogel under 400 nm excitation light. **(D)** Representative microscopic image of the cross-section of the microneedle showing the translucent hydrogel inside the microneedle's bore.

Figure S6: Fabrication of the detection device for glucose monitoring. (A) Schematic illustration of the 3D structure of the microneedle device. **(B)** 3D structure of the multifunctional chip. An optical sensor and a light resource are configured on a single chip. **(C)** Side view of the device compared to the Airpod case.

Figure S7: Microneedle device with the nano-diamond hydrogel can detect glucose *in vitro* and *in vivo*. (A) Changes of glucose concentration *in vitro* can be repeatedly measured by the device after 3 months of storage in ambient light. n = 3. Data are presented as mean ± SD. All error bars represent SD. **(B-C)** Similar as data in Fig. 4A and B, blood glucose level can be detected by a separate device with glucose-challenged (C) or insulin-challenged (D) mice. **(D-E)** Similar as data in Fig. 4C and D, after storage of, blood glucose level can be monitored with a different microneedle device after 3 months storage at ambient light, for glucose-challenged (D) or insulin-challenged (E) animals.

Figure S8: Fluorescence intensity detected by the nano-diamond based device can continuously trace glucose level even under strong magnetic field. The photos of the sensor (A), internal metal electronics (B), and magnetic attraction (C) of the Abbott FreeStyle Libre 2 continuous glucose monitoring system. The experimental setup without (D) and with (E) the magnet approached. (F) Notification popped up and the sensor did not function when the magnet approached. (G) Fluorescent nano-diamond

based sensor detected glucose level even under strong magnetic field, but not the Abbott FreeStyle Libre 2.

Figure S9: Fluorescence intensity detected by the nano-diamond based device can continuously trace glucose level under in low oxygen environments. (A) In PBS buffers, fluorescent nano-diamond boronic hydrogel based device and Abbott FreeStyle Libre 2 were able to reflect the glucose level. n = 4. Data are presented as mean ± SD. All error bars represent SD. (B) Placing the Libre 2 sensor from regular buffer into deoxygenated buffer with the same glucose concentration resulted in a decrease in the number of resulting glucose concentrations and the detection function quickly became unusable. (C) Nano-diamond based device was able to detect the glucose level in deoxygenated PBS buffers. n = 4. Data are presented as mean ± SD. All error bars represent SD.

Figure S10: The fluorescence intensity detected by our device correlates with glucose concentrations in vitro over the physiological temperature values. (A) Sensor dysfunction after rapid change in ambient temperature. (B) Nano-diamond based device can monitor the glucose level change in water bath at 30 °C, 37 °C, and 42 °C. n = 4. Data are presented as mean ± SD. All error bars represent SD.

Figure S11: Sections of the skin after microneedle insertion or full-thickness skin wounds (3 mm) were stained with Trichrome, F4/80 or CD3 antibodies. Red dashed lines denote lesion area.

Figure S12: Another representative result of glucose monitoring with porcine skin model.

Figure S1

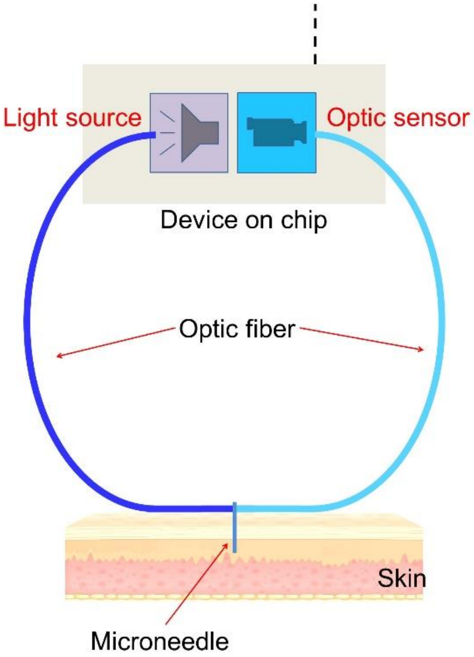

Light source
Optic sensor
Device on chip
Optic fiber
Skin
Microneedle

Figure S2

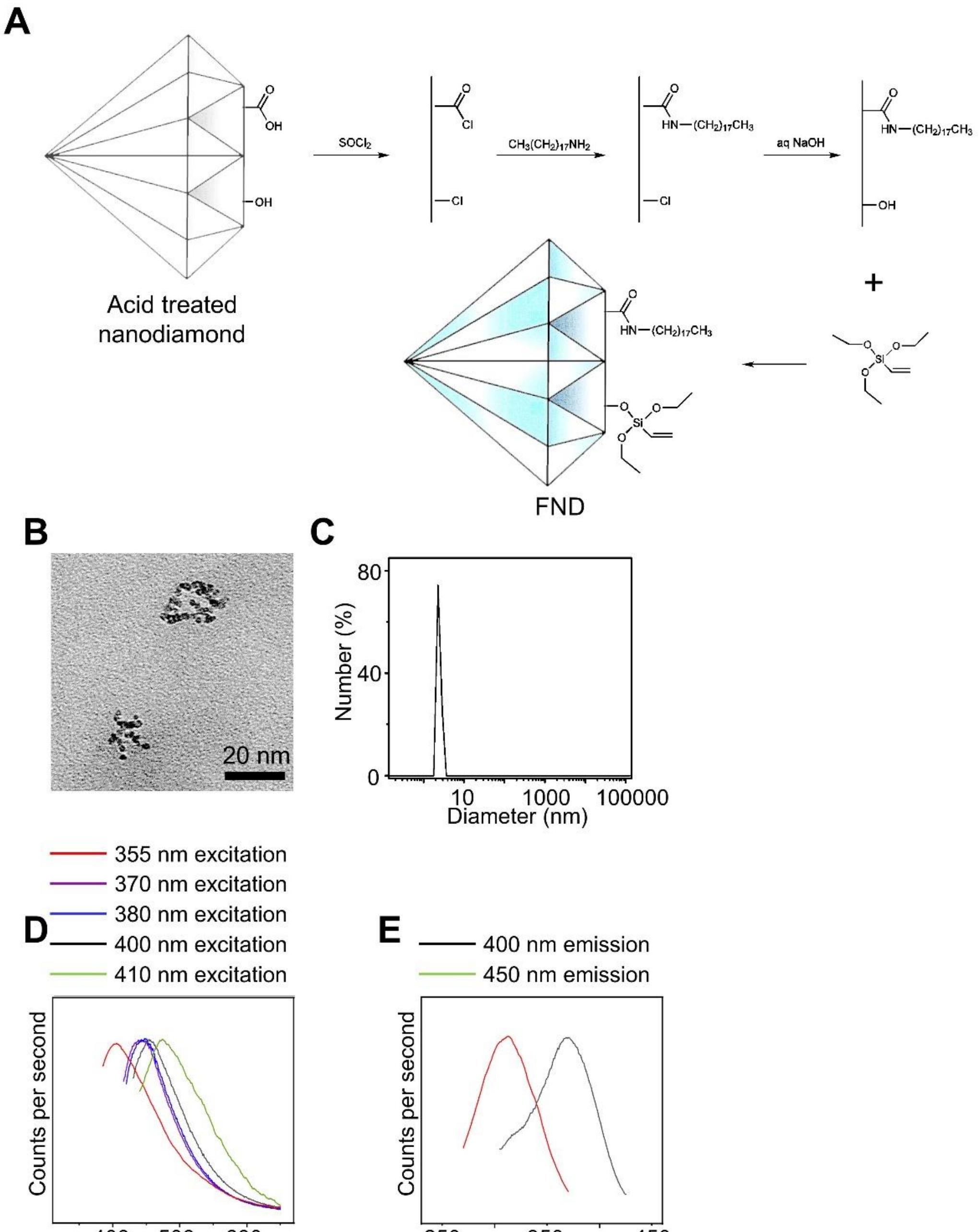

A
SOCl2
CH3(CH2)17NH2
aq NaOH
Acid treated nanodiamond
FND
B
20 nm
C
Number (%)
Diameter (nm)
D
355 nm excitation
370 nm excitation
380 nm excitation
400 nm excitation
410 nm excitation
Counts per second
Wavelength (nm)
E
400 nm emission
450 nm emission
Counts per second
Wavelength (nm)

Figure S3

A

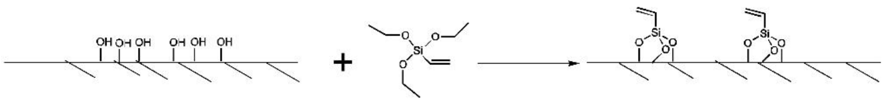

B

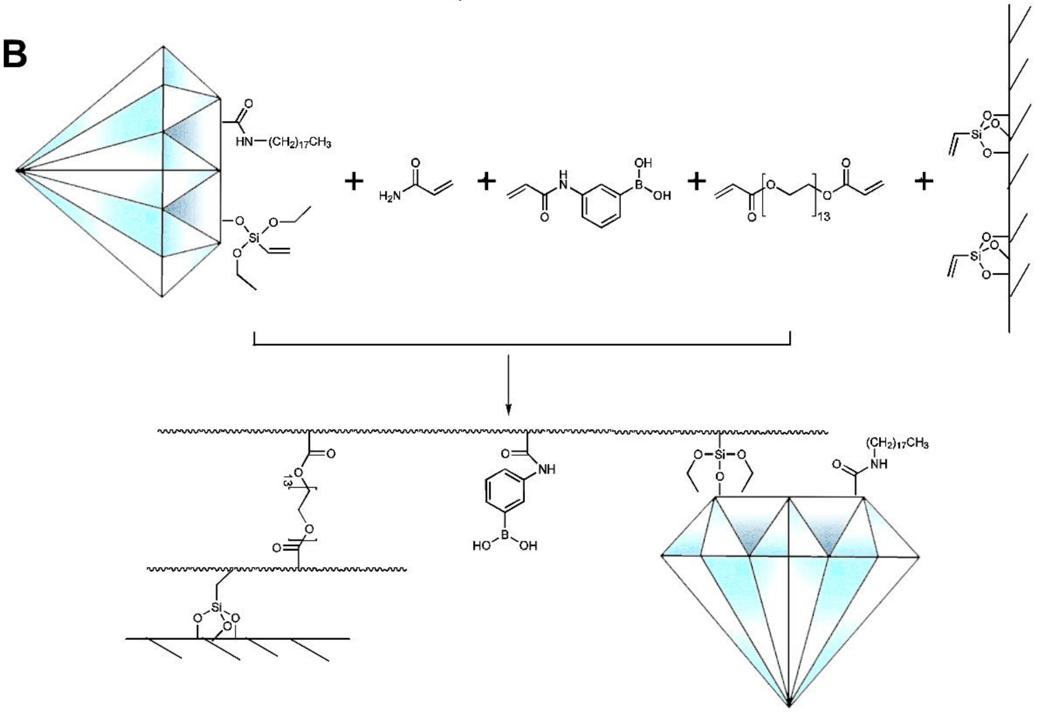

C

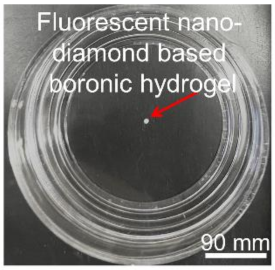

Figure S4

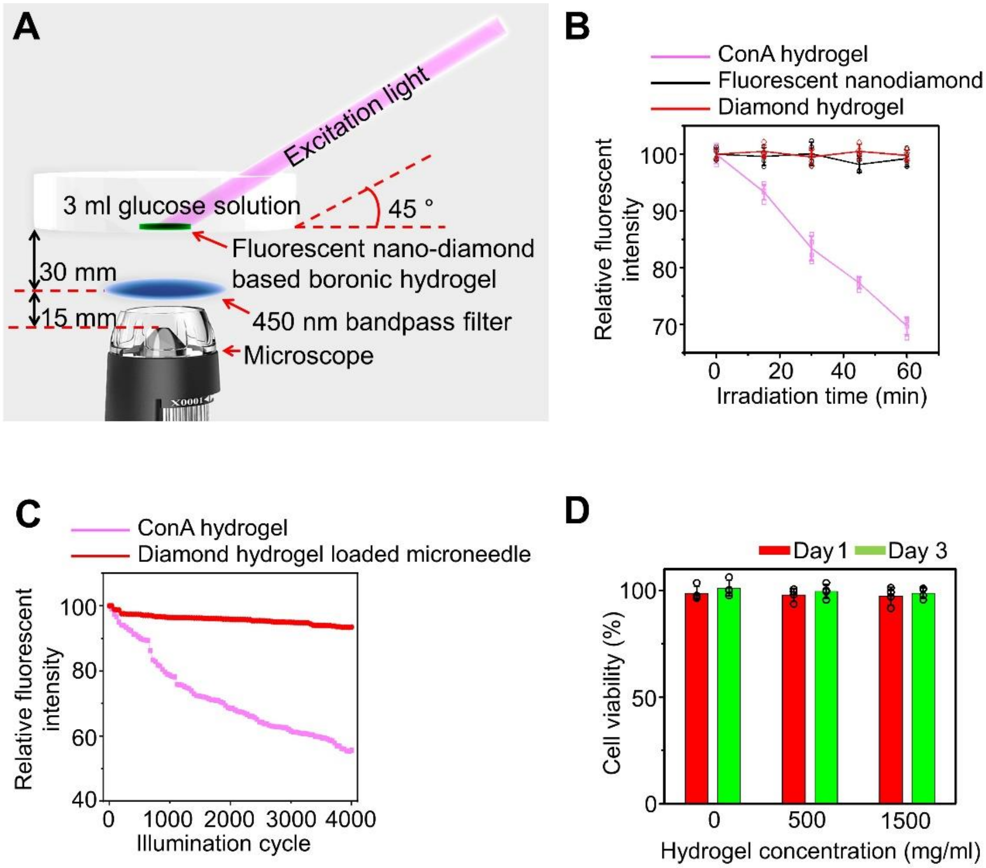
A
Excitation light
3 ml glucose solution
45 °
Fluorescent nano-diamond
based boronic hydrogel
30 mm
15 mm
450 nm bandpass filter
Microscope
B
ConA hydrogel
Fluorescent nanodiamond
Diamond hydrogel
Relative fluorescent intensity
Irradiation time (min)
C
ConA hydrogel
Diamond hydrogel loaded microneedle
Relative fluorescent intensity
Illumination cycle
D
Day 1
Day 3
Cell viability (%)
Hydrogel concentration (mg/ml)

Figure S5

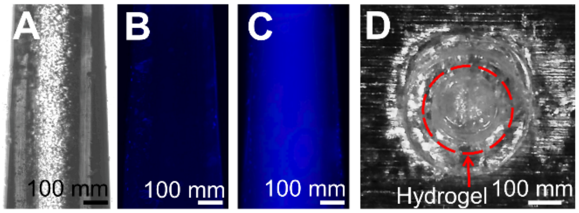

Figure S6

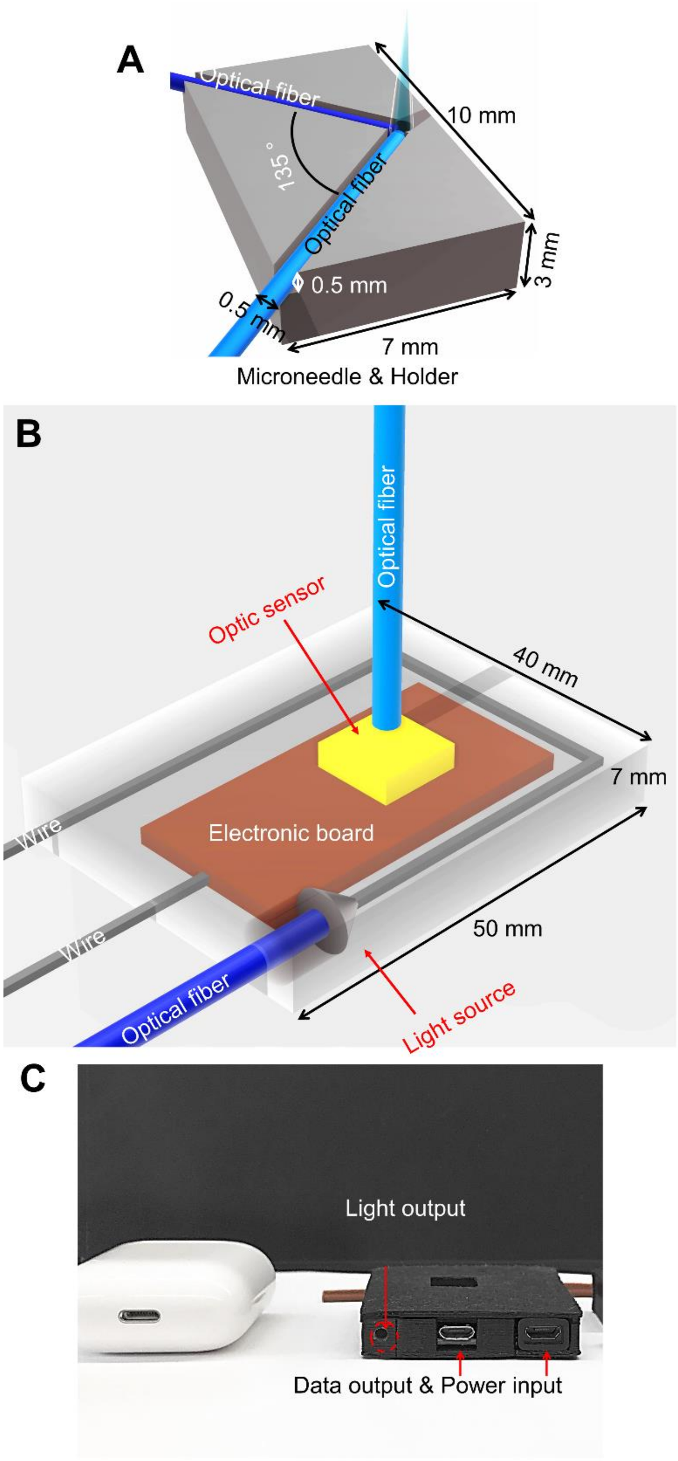
A
Optical fiber
135 °
Optical fiber
10 mm
3 mm
0.5 mm
0.5 mm
7 mm
Microneedle & Holder
B
Optical fiber
Optic sensor
40 mm
7 mm
Electronic board
Wire
Wire
50 mm
Optical fiber
Light source
C
Light output
Data output & Power input

Figure S7

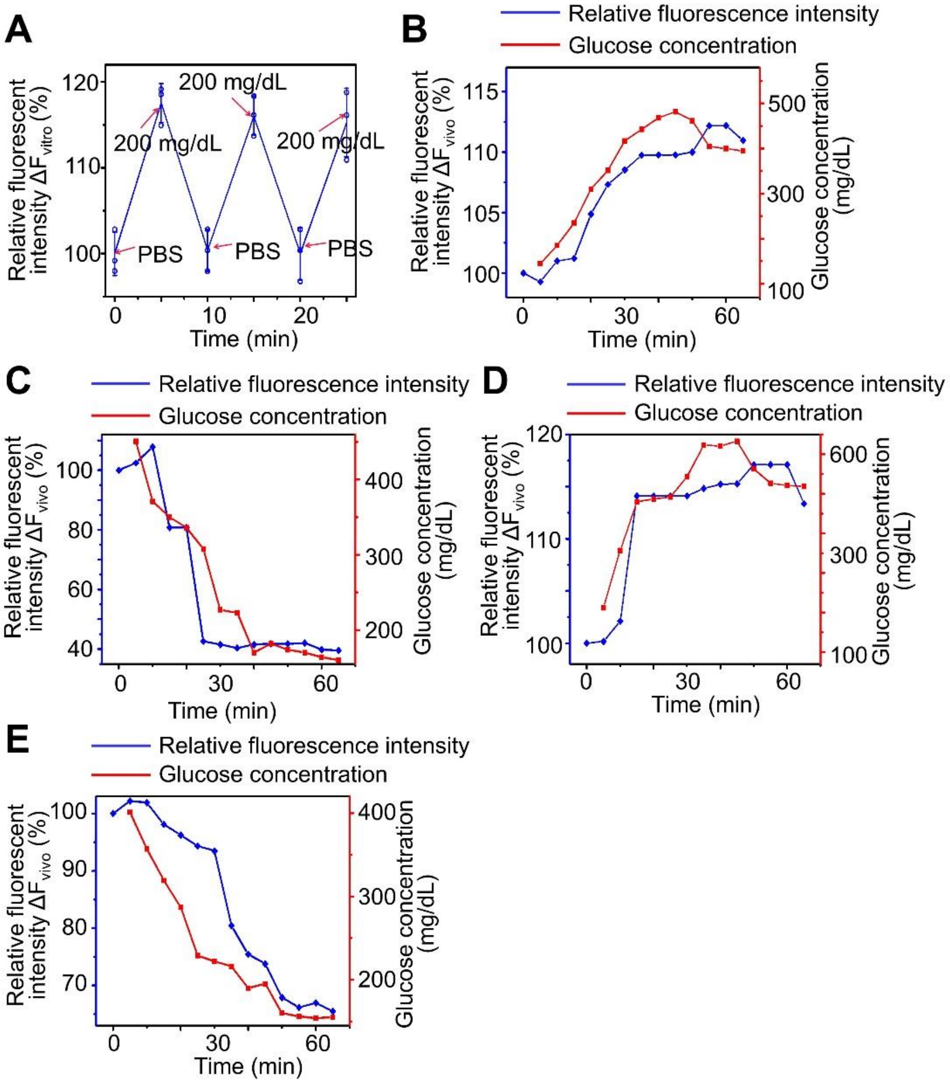
A
Relative fluorescent intensity ΔF_{vitro} (%)
Time (min)
200 mg/dL
200 mg/dL
200 mg/dL
PBS
PBS
PBS
B
Relative fluorescence intensity
Glucose concentration
Relative fluorescent intensity ΔF_{vivo} (%)
Glucose concentration (mg/dL)
Time (min)
C
Relative fluorescence intensity
Glucose concentration
Relative fluorescent intensity ΔF_{vivo} (%)
Glucose concentration (mg/dL)
Time (min)
D
Relative fluorescence intensity
Glucose concentration
Relative fluorescent intensity ΔF_{vivo} (%)
Glucose concentration (mg/dL)
Time (min)
E
Relative fluorescence intensity
Glucose concentration
Relative fluorescent intensity ΔF_{vivo} (%)
Glucose concentration (mg/dL)
Time (min)

Figure S8

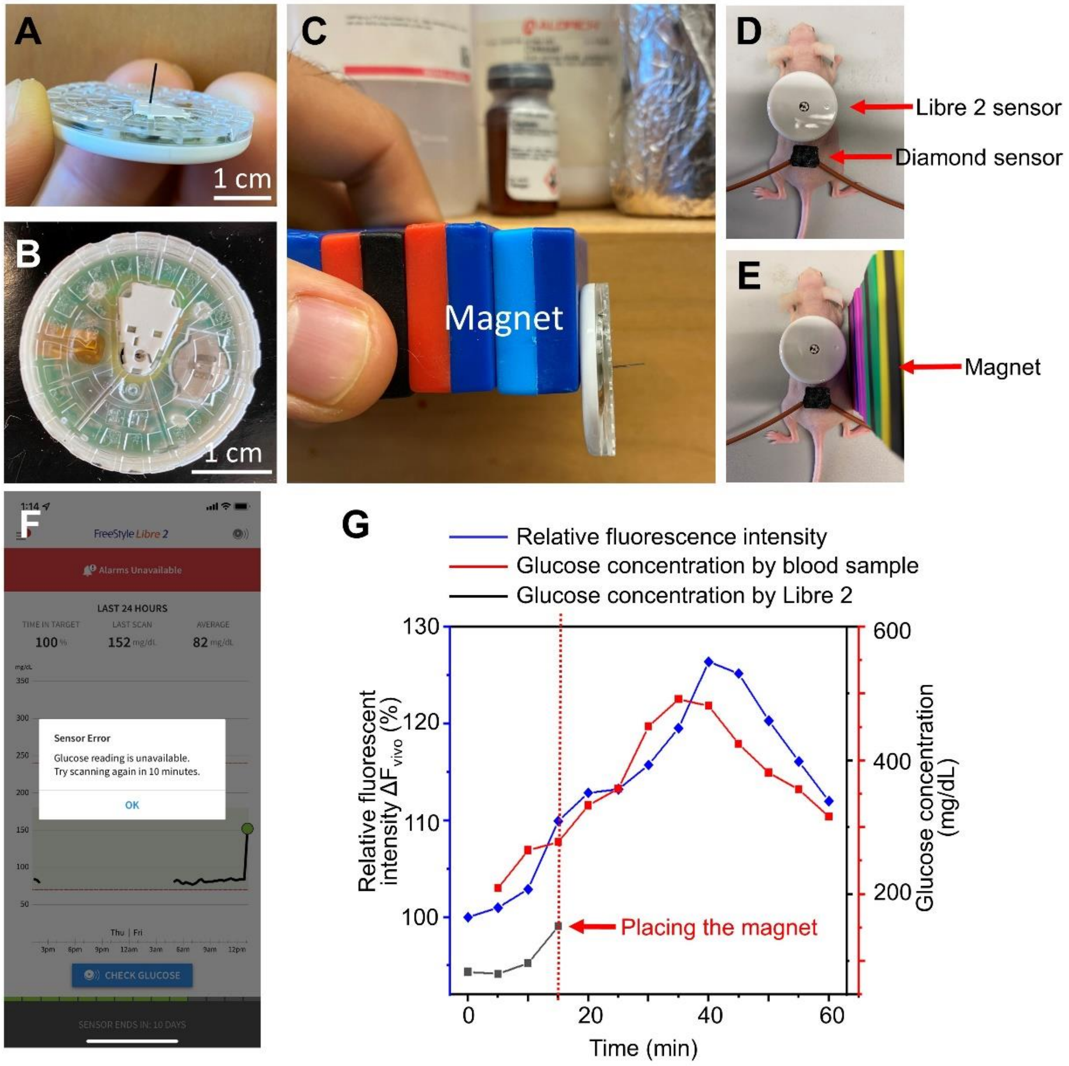
A
1 cm
B
1 cm
C
Magnet
D
Libre 2 sensor
Diamond sensor
E
Magnet
F
FreeStyle Libre 2
Alarms Unavailable
LAST 24 HOURS
TIME IN TARGET
LAST SCAN
AVERAGE
100 %
152 mg/dL
82 mg/dL
Sensor Error
Glucose reading is unavailable.
Try scanning again in 10 minutes.
OK
CHECK GLUCOSE
SENSOR ENDS IN: 10 DAYS
G
Relative fluorescence intensity
Glucose concentration by blood sample
Glucose concentration by Libre 2
Placing the magnet
Relative fluorescent intensity ΔF vivo (%)
Glucose concentration (mg/dL)
Time (min)

Figure S9

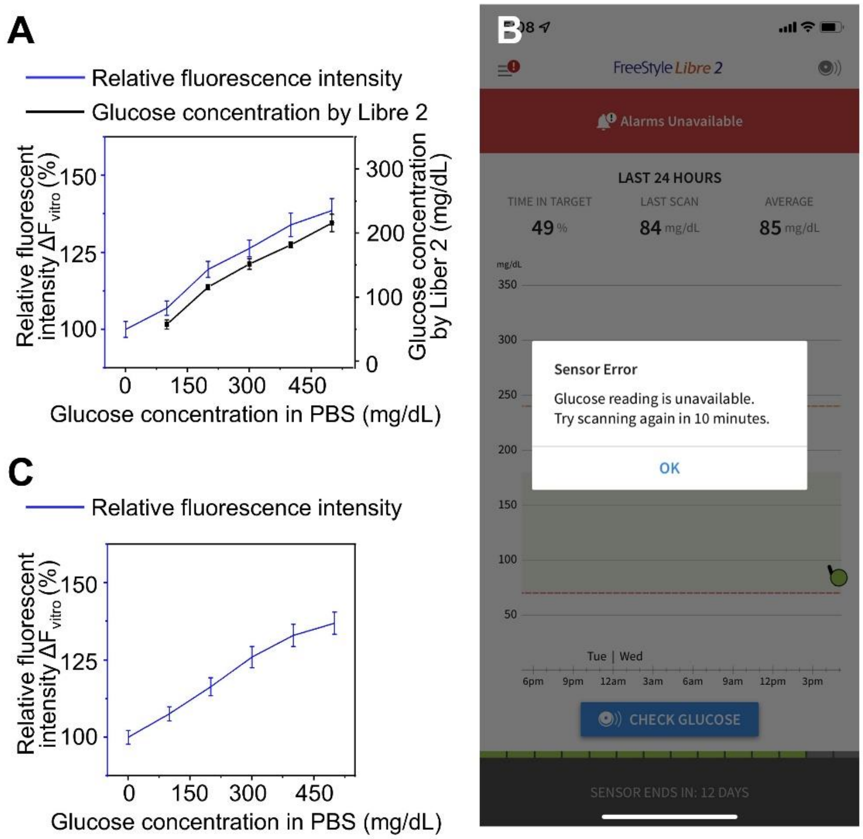
A
Relative fluorescence intensity
Glucose concentration by Libre 2
Relative fluorescent intensity ΔF vitro (%)
Glucose concentration by Liber 2 (mg/dL)
Glucose concentration in PBS (mg/dL)
B
FreeStyle Libre 2
Alarms Unavailable
LAST 24 HOURS
TIME IN TARGET
49 %
LAST SCAN
84 mg/dL
AVERAGE
85 mg/dL
Sensor Error
Glucose reading is unavailable.
Try scanning again in 10 minutes.
OK
CHECK GLUCOSE
SENSOR ENDS IN: 12 DAYS
C
Relative fluorescence intensity
Relative fluorescent intensity ΔF vitro (%)
Glucose concentration in PBS (mg/dL)

Figure S10

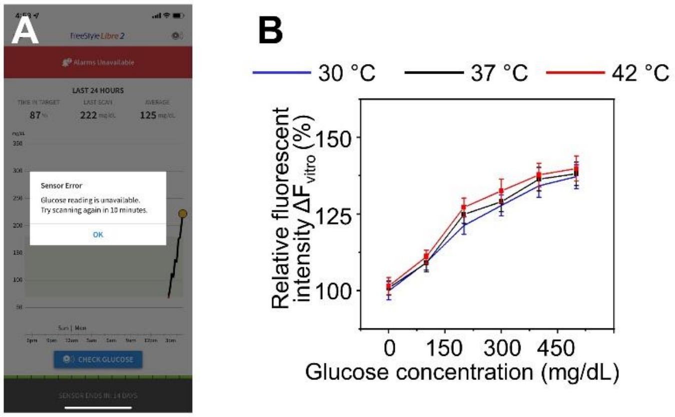

A
FreeStyle Libre 2
Alarms Unavailable
LAST 24 HOURS
87
222
125
Sensor Error
Glucose reading is unavailable.
Try scanning again in 10 minutes.
OK
CHECK GLUCOSE
B
30 °C
37 °C
42 °C
Relative fluorescent intensity ΔF_{vitro} (%)
150
125
100
0
150
300
450
Glucose concentration (mg/dL)

Figure S11

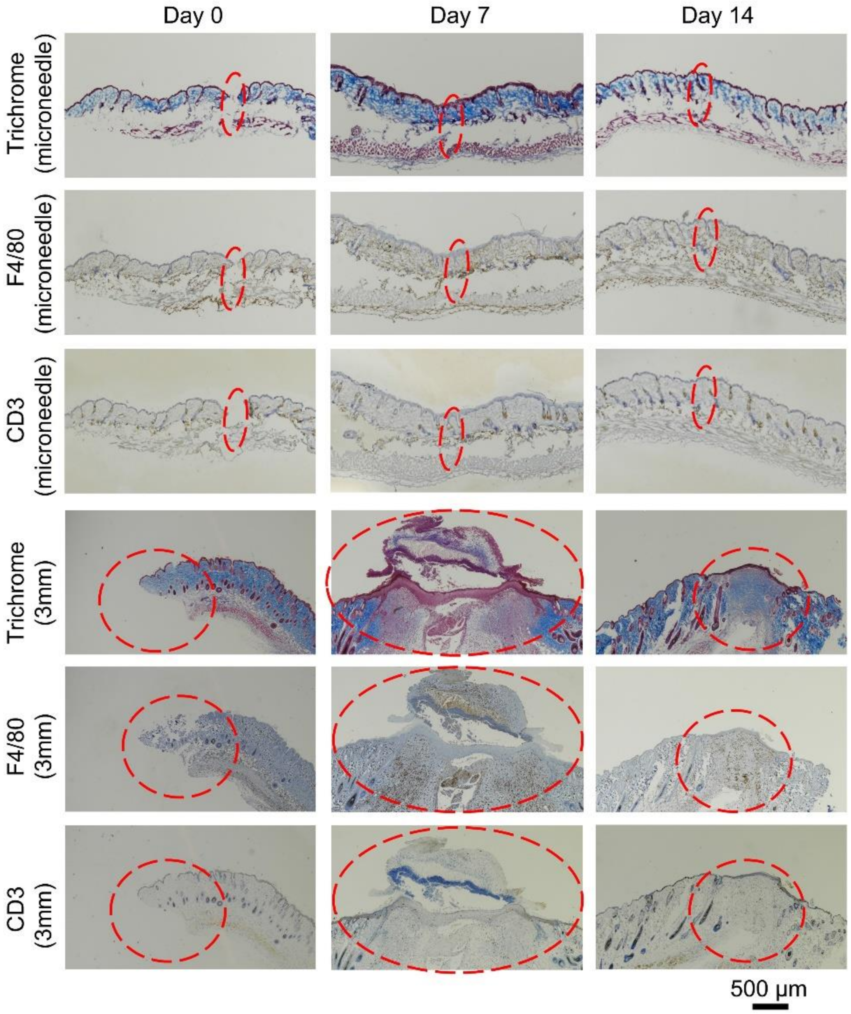
Day 0
Day 7
Day 14
Trichrome (microneedle)
F4/80 (microneedle)
CD3 (microneedle)
Trichrome (3mm)
F4/80 (3mm)
CD3 (3mm)
500 µm

Figure S12

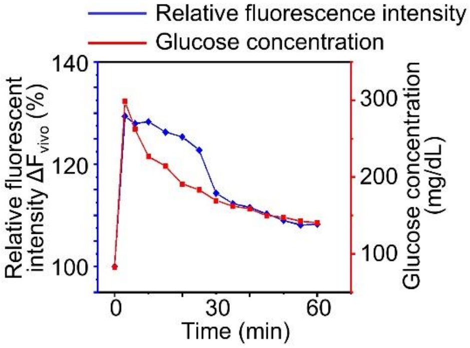

Relative fluorescence intensity
Glucose concentration
Relative fluorescent intensity ΔF vivo (%)
140
120
100
Glucose concentration (mg/dL)
300
200
100
0
30
60
Time (min)
